# THE MEANING OF FAMILYHOOD FOR GRAY DIVORCE IN ISRAEL

**DOI:** 10.1093/geroni/igac059.157

**Published:** 2022-12-20

**Authors:** Chaya Koren, Yafit Cohen, Naor Demeter, Michal Egert

**Affiliations:** University of Haifa, Haifa, Hefa, Israel; School of Social Work University of Haifa, Haifa, Hefa, Israel; School of Social Work University of Haifa, Haifa, Hefa, Israel; School of Social Work University of Haifa, Haifa, Hefa, Israel

## Abstract

Israel is a society that values familyhood alongside self-determination. Gray divorce rates in Israel are low, yet they have nearly doubled since 1996 from 2% to 3.65%. Little is known about grey divorce in modern societies and even less in societies located between tradition and modernity such as Israel. Deriving from data analysis, our aim is to present the meaning of familyhood for gray divorce in Israel from the experiences of individuals and couples who divorced at age 60+ and their adult children, using a life course perspective. 72 in-depth qualitative interviews were audio recorded and transcribed verbatim with divorced men, women, and their adult children, analyzed as individuals, dyads, and family units based on principles of dyadic interview analysis. Findings include three themes: (a) The value of familyhood as a divorce delay. (b) Between couplehood dissolution and familyhood preservation. (c) Gray divorce shaping a new familyhood. Implications are discussed.

